# Intersection of Coagulation and Fibrinolysis by the Glycosylphosphatidylinositol (GPI)-Anchored Serine Protease Testisin

**DOI:** 10.3390/ijms24119306

**Published:** 2023-05-26

**Authors:** Marguerite S. Buzza, Nisha R. Pawar, Amando A. Strong, Toni M. Antalis

**Affiliations:** 1 Center for Vascular and Inflammatory Diseases, University of Maryland School of Medicine, Baltimore, MD 21201, USA; npawar@som.umaryland.edu (N.R.P.); amando.strong@som.umaryland.edu (A.A.S.); tantalis@som.umaryland.edu (T.M.A.); 2 Department of Physiology, University of Maryland School of Medicine, Baltimore, MD 21201, USA; 3 Marlene and Stewart Greenebaum Comprehensive Cancer Center, University of Maryland School of Medicine, Baltimore, MD 21201, USA; 4 Research and Development Service, VA Maryland Health Care System, Baltimore, MD 21201, USA

**Keywords:** serine protease, cell surface, hemostasis, fibrinolysis, coagulation factor, testisin

## Abstract

Hemostasis is a delicate balance between coagulation and fibrinolysis that regulates the formation and removal of fibrin, respectively. Positive and negative feedback loops and crosstalk between coagulation and fibrinolytic serine proteases maintain the hemostatic balance to prevent both excessive bleeding and thrombosis. Here, we identify a novel role for the glycosylphosphatidylinositol (GPI)-anchored serine protease testisin in the regulation of pericellular hemostasis. Using in vitro cell-based fibrin generation assays, we found that the expression of catalytically active testisin on the cell surface accelerates thrombin-dependent fibrin polymerization, and intriguingly, that it subsequently promotes accelerated fibrinolysis. We find that the testisin-dependent fibrin formation is inhibited by rivaroxaban, a specific inhibitor of the central prothrombin-activating serine protease factor Xa (FXa), demonstrating that cell-surface testisin acts upstream of factor X (FX) to promote fibrin formation at the cell surface. Unexpectedly, testisin was also found to accelerate fibrinolysis by stimulating the plasmin-dependent degradation of fibrin and enhancing plasmin-dependent cell invasion through polymerized fibrin. Testisin was not a direct activator of plasminogen, but it is able to induce zymogen cleavage and the activation of pro-urokinase plasminogen activator (pro-uPA), which converts plasminogen to plasmin. These data identify a new proteolytic component that can regulate pericellular hemostatic cascades at the cell surface, which has implications for angiogenesis, cancer biology, and male fertility.

## 1. Introduction

The trypsin-like serine proteases comprise a large family of proteolytic enzymes with diverse biological functions including blood coagulation, fibrinolysis, food digestion, and fertilization. These proteases are defined by a conserved catalytic domain containing a catalytic triad of histidine, aspartate, and serine amino acid residues that form their active site and mediate the process of peptide hydrolysis. Most of the well-characterized trypsin-like proteases such as trypsin, thrombin, plasmin, or the plasminogen activators are soluble proteases that are secreted into the extracellular environment. There exists a unique sub-group of trypsin-like serine proteases that are anchored in the cell membrane, either by a transmembrane domain at the N- or C-terminus or via a glycosylphosphatidylinositol (GPI) anchor, which collectively are known as membrane-anchored serine proteases (MASPs). Like soluble serine proteases, they are synthesized as inactive zymogens and possess the ability to cross-activate and reciprocally activate other protease zymogens [1,2,3]. However, the specific functions of many of these proteases, as well as their substrates and pathophysiological functions, are poorly understood.

Testisin, also known as PRSS21, is one of two human GPI-anchored serine proteases [1,2,4]. Testisin was originally characterized in the developing spermatocytes of the testis [4,5], where it is not secreted but found on the cell surface localized to specialized membrane microdomains known as lipid rafts [4,5,6]. The complete genetic deletion of testisin in *Prss21^−/−^* mice causes defective epidydimal sperm maturation and reduced fertilizing ability compared to *Prss21^+/+^* littermate control mice [7,8,9]. Beyond sperm, testisin expression is extremely restricted in healthy individuals, being expressed at low levels in endothelial cells and some immune cells [4,10,11,12,13], and it is overexpressed in ovarian cancers compared to normal ovary tissue [14,15]. Using a protease-gene-specific screen, we identified testisin expression in human dermal microvascular endothelial cells (HMVECs) undergoing reorganization and tubule-like formation on the basement membrane Matrigel, as well as during pre-capillary morphogenesis on fibrillar type I collagen [10]. A global microarray analysis of human endothelial cell diversity in 14 distinct vascular tissues also identified testisin as expressed consistently in both large vessels and the microvasculature [16]. In vitro siRNA knockdown studies showed that the loss of testisin markedly impairs microvascular endothelial cell migration and reorganization during Matrigel-induced angiogenesis [12]. In a recent study of the effect of testisin deletion on angiogenesis during corpus luteal development, we found that *Prss21^−/−^* mice display a strikingly increased incidence and severity of hemorrhages, which were associated with impaired endothelial barrier formation and function [12].

Testisin and the other MASPs are S1 family serine proteases that share several features with the serine proteases of the coagulation and fibrinolytic systems [1,2,3]. Fibrin deposition and remodeling are prominent features of spermatogenesis and angiogenesis. Seminal fluid contains coagulation and fibrinolytic proteases that are believed to facilitate the coagulation and subsequent liquification of semen after ejaculation [17]. During repair-associated angiogenesis, a provisional fibrin matrix acts not only as a sealing matrix, but also as a scaffolding for invading endothelial cells [18]. The deposition of fibrin occurs as a result of the cleavage of circulating fibrinogen by the serine protease thrombin, to induce its polymerization into cross-linked fibrin networks [19,20]. Thrombin is produced by the proteolytic activation of the prothrombin zymogen by Factor X (FXa), which is produced via either the extrinsic or intrinsic coagulation cascades [21,22]. The persistence of fibrin is regulated by the fibrinolytic system, which is activated simultaneously with the coagulation cascade and functions to degrade fibrin. Plasmin, the key protease that directly mediates the degradation of fibrin [23,24], is generated from plasminogen by the plasminogen activator’s tissue-type plasminogen activator (tPA) and urokinase plasminogen activator (uPA). tPA plays a significant role in removing fibrin from the vascular tree and maintaining vascular patency, whereas uPA and its receptor urokinase-type plasminogen activator receptor (uPAR) are important mediators of pericellular proteolysis required for cell migration and tissue remodeling during wound healing [25]. uPA is expressed by angiogenic endothelium, and its expression and activity are often upregulated by tumor cells, where it facilitates tumor cell migration and metastasis [23,26,27].

We hypothesized that cell surface testisin could be uniquely localized to intersect hemostatic pathways that regulate fibrin deposition and/or remodeling on the cell surface in the pericellular microenvironment. Here, we present evidence for two novel activities of membrane-anchored testisin: (1) the acceleration of FXa-prothrombin-dependent fibrin generation; and (2) the activation of plasmin(ogen)-dependent fibrinolysis.

## 2. Results

### 2.1. Testisin Accelerates Fibrin Generation and Its Subsequent Degradation

**Figure 1:**
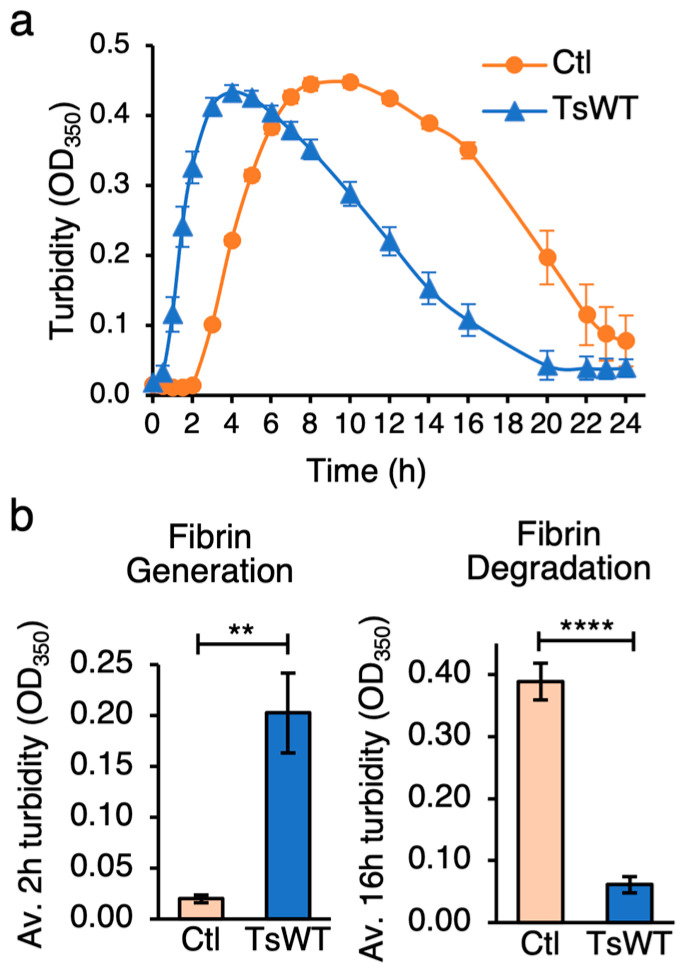
GPI-anchored testisin promotes fibrin generation and degradation. (**a**) Time course analysis of cell-based fibrin generation. TsWT and Ctl cells were treated with 1 mg/mL fibrinogen and 10 nM prothrombin zymogen at 0 h and turbidity at OD_350_ monitored over 24 h. Graph shows a representative experiment of average turbidity ± SEM from quadruplicate wells at the indicated times. (**b**) TsWT cells significantly accelerate both fibrin generation and its subsequent degradation compared to Ctl cells. Graphs show average turbidity measurements ± SEM at 2 h (**left** panel) and 16 h (**right** panel) from 5 independent experiments. ** *p* < 0.01; **** *p* < 0.001.

A cell-based functional assay was developed to measure fibrin generation on the cell surface. Adherent cells are provided with fibrinogen and the prothrombin zymogen, and the conversion of fibrinogen to fibrin is measured by an increase in turbidity resulting from the polymerization of fibrin [28]. Once formed, fibrinolysis can be detected by a subsequent decrease in turbidity as polymerized fibrin is degraded [28,29]. Cells that do not express significant levels of testisin were stably transfected to express full-length GPI-anchored testisin (TsWT) or vector alone (Ctl) [14] and characterized for testisin protein expression (Figure S1a). Cell-surface testisin activity was measured using the fluorogenic substrate Boc-QAR-AMC (Figure S1b). These cells were incubated with fibrinogen together with the prothrombin zymogen, and changes in turbidity were monitored spectrophotometrically over time. Testisin-expressing TsWT cells displayed a rapid increase in turbidity beginning within the first hour, indicative of a rapid rise in fibrin generation, which transiently peaked at around 4 h (Figure 1a), followed by a rapid decrease, indicating cell-mediated dissolution of the formed fibrin polymers. By comparison, there was a notable lag and a slower increase in turbidity by Ctl cells, which plateaued between 8–10 h (Figure 1a), and then decreased more slowly over 24 h. The delayed appearance of turbidity in Ctl cells indicates that the parental cells are capable of inducing prothrombin-dependent fibrin generation less efficiently than the testisin-expressing TsWT cells, indicating the possible presence of a secondary testisin-independent mechanism for fibrin generation by these cells. Quantitation of average turbidity measurements from independent experiments showed that compared to Ctl cells, testisin-expressing TsWT cells significantly accelerated fibrin generation (Figure 1b, left panel, 2 h turbidity) as well as fibrin degradation (Figure 1b, right panel, 16 h turbidity).

**Figure 2:**
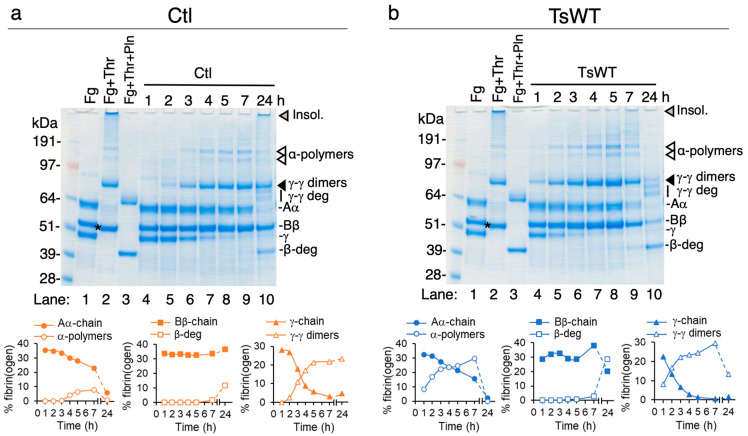
GPI-anchored testisin accelerates fibrin polymerization and degradation. Urea-solubilized lysates prepared from cell-based fibrin generation experiments from Ctl cells (**a**) and TsWT cells (**b**) were analyzed by reducing SDS-PAGE and Coomassie blue staining for detection of fibrinogen and fibrin species over 24 h. For reference, cell-free control wells that contain fibrinogen alone (Fg, lane 1) or polymerized fibrin (Fg + Thr, lane 2) and polymerized fibrin treated with plasmin (Fg + Thr + Pln, lane 3, generated by treatment of polymerized fibrin with 20 nM plasminogen and 2 nM uPA) are included. As reported [30], commercial preparations of fibrinogen contain trace amounts of contaminating FXIII, which covalently crosslinks fibrin monomers into insoluble fibrin polymers [19], so that the conversion of fibrinogen to fibrin by thrombin results in higher-molecular weight cross-linked species and the loss of the fibrinogen monomer α- and γ-chains (**a**,**b**, lane 1 vs. lane 2, arrow heads). A small decrease in molecular mass of the Bβ-chain also occurs due to the removal of fibrinopeptide B (**a**,**b**, lane 2, asterisk). The molecular masses of unpolymerized fibrinogen subunits (Aα, ~62 kDa; Bβ, ~48 kDa; γ, ~52 kDa), those of polymerized fibrin (γ-γ dimers, α-polymers, and the thrombin-cleaved β-chain), and those of fibrin that has been degraded by plasmin (γ-γ deg and β-deg) are shown. Fibrinogen/fibrin species and fibrin degradation products were identified as reported in [31,32]. Quantification of unpolymerized fibrinogen chain monomers and fibrin/fibrin degradation species by densitometry are shown below each gel. Levels of individual species are expressed as the % of the total fibrin(ogen) bands quantified in each sample.

To verify that the turbidity changes observed over time in the cell-based fibrin generation assays represent fibrin polymerization and subsequent degradation, urea-solubilized lysates from TsWT and Ctl cells collected over the time course were analyzed by reducing SDS-PAGE and Coomassie blue staining to visualize fibrinogen, crosslinked fibrin, and fibrin degradation products (Figure 2). Using densitometry, the intensity of fibrin(ogen) species was represented as a percentage of total fibrinogen in each lane (plots below each gel). A comparison of fibrinogen products from Ctl (Figure 2a) vs. TsWT (Figure 2b) shows an accelerated increase in the conversion of fibrinogen to fibrin by TsWT cells during the first 7 h (lanes 4–9), demonstrated by the faster loss of the γ-chain monomer, the appearance of γ-γ dimers (black arrowheads), and the appearance of α-polymers (white arrowheads) compared to Ctl cells, consistent with the increase in turbidity (Figure 1). Enhanced fibrin degradation by TsWT compared to Ctl cells was observed at 24 h, evidenced by decreased γ-γ dimers, enhanced generation of the β-chain degradation product (β-deg), the appearance of γ-chain degradation products, and the complete loss of α-polymers and urea-insoluble fibrin species (grey arrowheads), which remain present in Ctl lysates extending to 24 h (Figure 2a,b). The generated fibrin degradation products are of similar size to those that are produced when fibrin is treated with plasmin (Pln) (Figure 2a,b, lanes 3). Together, these data indicated that cell-expressed GPI-anchored testisin could both stimulate fibrin generation and accelerate subsequent fibrin degradation.

### 2.2. Catalytically Active Cell Surface Testisin Is Required for Fibrin Polymerization

**Figure 3:**
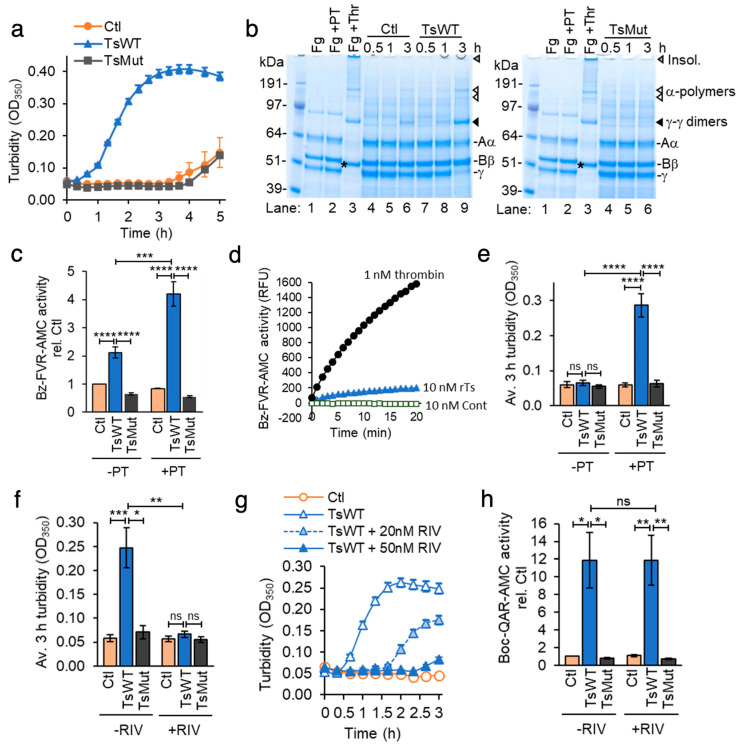
Catalytically active testisin accelerates prothrombin-dependent fibrin generation through a FXa-dependent mechanism. (**a**) Testisin-induced fibrin generation is dependent upon its catalytic activity. TsWT, TsMut, and Ctl cells were treated with 1 mg/mL fibrinogen with 10 nM prothrombin zymogen and turbidity at OD_350_ monitored over 5 h. Graph shows average OD_350_ from quadruplicate wells ± SEM and is representative of at least 5 independent experiments. (**b**) SDS-PAGE of urea-solubilized lysates confirms faster generation of fibrin in TsWT cells compared to Ctl and TsMut cells. Lysates taken at 0.5, 1, and 3 h from the experiment (**a**) stained with Coomassie blue are shown. Shown also are γ-γ dimers (black arrows), monomer fibrinogen γ-chain, α-polymers (white arrows), and the thrombin-cleaved β-chain (*). Control lanes show cell-free fibrinogen alone (Fg, lane 1), fibrinogen and prothrombin (Fg + PT, lane 2), and polymerized fibrin (Fg + Thr, lane 3). (**c**) TsWT cells stimulate prothrombin activation. Thrombin activity was measured using the Bz-FVR-AMC fluorogenic substrate on cells treated with fibrinogen alone (−PT) or with 10 nM prothrombin (+PT) and fluorescence measured at Ex370 nm/Em450 nm. Graph shows average thrombin activity normalized to Ctl + Fg alone ± SEM at 3 h from 4–6 independent experiments. (**d**) Compared to thrombin, testisin catalyzes only a low level of proteolytic cleavage of the fluorogenic thrombin substrate Bz-FVR-AMC. Activity (relative fluorescence units, RFU) of α-thrombin (1 nM), rTs (10 nM) or rTs control (Cont., (10 nM) see Materials and Methods) was measured from duplicate wells over 20 min. Graph is representative of 4 independent experiments. (**e**) Average turbidity measurements at 3 h from 5–6 independent experiments in Ctl, TsWT, and TsMut cells in the presence of fibrinogen, with (+) or without (−) 10 nM prothrombin (PT). (**f**) Rivaroxaban does not inhibit cell-expressed testisin. Testisin activity was assayed in the presence of 1 mg/mL fibrinogen with (+) or without (−) 50 nM rivaroxaban using the Boc-QAR-AMC substrate. Graph shows average testisin activity at 3 h ± SEM from 3 independent experiments. Data are normalized to Ctl + Fg alone. (**g**) Rivaroxaban dose-dependently inhibits prothrombin-dependent induction of turbidity mediated by TsWT cells. Turbidity assay in the presence of fibrinogen and prothrombin in which TsWT cells were treated with 20 or 50 nM rivaroxaban or no inhibitor. Turbidity was monitored over 3 h. Graph represents mean ± SEM from triplicate wells. (**h**) Average turbidity induced by Ctl, TsWT and TsMut cells in the absence (−) or presence (+) of 50 nM rivaroxaban (RIV) at 3 h ± SEM from 3–4 independent experiments. * *p* < 0.05; ** *p* < 0.01; *** *p* < 0.005; **** *p* < 0.001; ns, non-significant.

To investigate the requirement for cell-surface testisin catalytic activity in the cell-based fibrin generation assay, we tested the ability of cells expressing the catalytically inactive S238A testisin mutant (TsMut) to stimulate fibrin generation. TsMut cells have been described previously [14] and express comparable levels of testisin protein by immunoblot analysis (Figure S1a). Testisin cell-surface activity is abrogated in these cells compared to TsWT as measured using the fluorogenic Boc-QAR-AMC substrate (Figure S1b). The turbidity change seen with TsWT cells is abrogated in cells expressing TsMut, which show a lag similar to Ctl cells (Figure 3a), demonstrating that the observed acceleration of fibrin generation requires testisin proteolytic activity. An analysis of solubilized lysates taken over the time course of 0.5–3 h from TsWT, TsMut, and Ctl cells after the addition of fibrinogen (Fg) and prothrombin shows that fibrinogen is converted into fibrin more rapidly by TsWT cells compared to TsMut cells (Figure 3b), demonstrated by the appearance of γ-γ dimers (Figure 3b, black arrow heads) and α-polymers (Figure 3b, white arrow heads) within 3 h and the loss of the γ-chain monomer (Figure 3b, γ) in TsWT cells compared to TsMut cells. These data demonstrate that catalytically active testisin on the cell membrane is required to enhance the rate of fibrin polymerization.

### 2.3. Fibrin Generation by Cell Surface Testisin Is Prothrombin-Dependent

Fibrin polymerization is catalyzed by the cleavage of fibrinogen by thrombin, produced by proteolytic activation of the prothrombin zymogen. To investigate the possibility that testisin may possess thrombin-like catalytic activity capable of direct fibrinogen cleavage, cell-surface thrombin activity of TsWT, TsMut, and Ctl cells was measured using the fluorogenic thrombin substrate Bz-FVR-AMC. In the presence of prothrombin, thrombin activity was significantly enhanced by TsWT cells (Figure 3c, +PT), which was not observed with Ctl or TsMut cells (Figure 3c, +PT). In the absence of prothrombin, TsWT cells display a low level of thrombin-like activity, which was higher than that of Ctl and TsMut cells (Figure 3c, −PT). Assays of thrombin activity using recombinant α-thrombin and recombinant testisin shows that testisin weakly recognizes this substrate (Figure 3d), which likely explains the low level of thrombin-like activity observed in the absence of prothrombin. When the cell-based fibrin generation assay was performed in the presence or absence of prothrombin, we found that after 3 h in the presence of prothrombin, TsWT cells induced a significant ~5-fold increase in turbidity compared to Ctl and TsMut cells (Figure 3e, +PT), with no increase in turbidity in the absence of prothrombin (Figure 3e, −PT), demonstrating that the testisin-induced fibrin generation requires the presence of prothrombin. Together, these data show that membrane-anchored testisin accelerates prothrombin-dependent activation on the cell surface, leading to fibrin polymerization.

### 2.4. Membrane-Anchored Testisin Activates a FXa-Dependent Pathway to Stimulate Fibrin Generation

At the convergence of the intrinsic and extrinsic coagulation pathways, activated Factor Xa catalyzes the conversion of prothrombin to thrombin as part of the prothrombinase complex [22]. To investigate whether testisin may catalyze the activation of prothrombin directly or function upstream of FXa, we utilized the specific and direct FXa inhibitor rivaroxaban (RIV, BAY 59-7939) [33,34]. RIV is highly specific for the inhibition of FXa [33] and has no direct inhibitory activity against recombinant testisin (Figure S2a) or cell-expressed, membrane-anchored testisin (Figure 3f and Figure S2b). When the cell-based fibrin generation assay was performed in the presence or absence of RIV, we found that RIV inhibited the ability of TsWT cells to induce prothrombin-dependent induction of turbidity in a dose-dependent manner (Figure 3g). RIV at a clinically relevant concentration (50 nM) completely blocked testisin-induced turbidity (Figure 3h) and inhibited the formation of polymerized fibrin, as visualized by reducing SDS-PAGE and Coomassie blue staining (Figure S2c). These data indicate that testisin does not directly activate prothrombin but acts upstream of FXa to induce prothrombin-dependent fibrin generation.

FXa is produced by activation cleavage of the FX two-chain disulfide-bond-linked zymogen at the Arg194↓Ile195 bond, releasing a highly glycosylated 52-amino acid activation peptide and the acquisition of enzymatic activity [35]. We did not find evidence for testisin activation cleavage of the FX zymogen by incubation of the FX zymogen with recombinant testisin (Figure S3a), nor could we detect the induction of FXa activity after incubation of the FX zymogen with recombinant testisin, as measured using the FXa substrate Boc-IEGR-AMC [36] (Figure S3b). These data suggest that testisin does not directly activate FX to produce FXa but instead acts upstream of FXa to induce prothrombin-dependent fibrin generation.

### 2.5. Testisin Acceleration of Fibrin Degradation Is Plasmin(ogen)-Dependent

**Figure 4:**
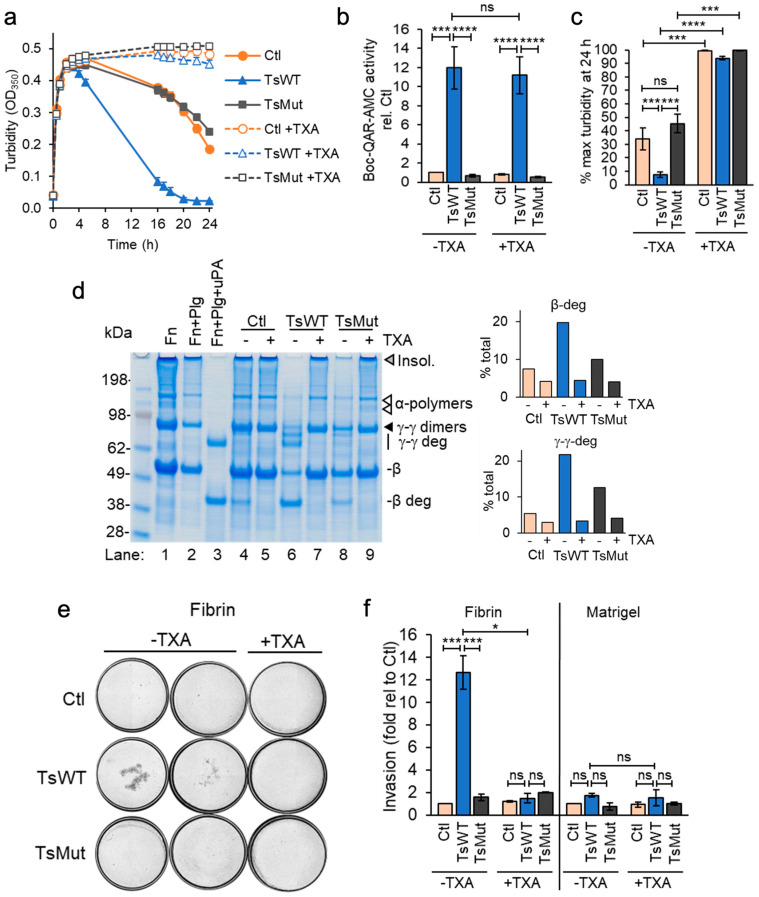
Acceleration of fibrinolysis by GPI-anchored testisin is dependent upon plasmin (ogen). (**a**) Time course analysis of cell-based fibrinolysis assays. TsWT, TsMut, and Ctl cells were treated with fibrinogen, without (closed symbols) or with 250 µM TXA (open symbols) and fibrin polymerization initiated with thrombin. Turbidity was monitored over 24 h. Data are representative of 3 independent experiments and show average turbidity ± SEM of quadruplicate wells. (**b**) TXA does not inhibit the activity of cell-expressed testisin, assessed in the presence of 1 mg/mL fibrinogen with (+) or without (−) 250 µM TXA using the peptide substrate Boc-QAR-AMC. Graph shows average activity ± SEM from 5 independent experiments at 3 h after adding substrate and is normalized to Ctl -TXA. (**c**) Average turbidity at the 24 h endpoint in the absence (−) or presence (+) of 250 µM TXA from 3 independent experiments. Data are expressed as % maximal turbidity reached after addition of thrombin in each experimental treatment. (**d**) SDS-PAGE confirms faster fibrin degradation by TsWT cells compared to Ctl and TsMut. Urea-solubilized lysates prepared at the 24 h endpoint analyzed by reducing SDS-PAGE stained with Coomassie blue. Control lanes include fibrin alone (Fn, lane 1), fibrin generated in the presence of 20 nM plasminogen (Fn + Plg, lane 2), and fibrin generated in the presence of plasminogen to which 5 nM uPA was added after polymerization to generate plasmin (Fn + Plg + uPA, lane 3). Fibrin degradation is shown by loss of insoluble fibrin (gray triangle) and α-polymers (white triangles). γ-γ dimers (black triangle) and the β-chain are also cleaved, producing γ-γ deg and β-deg fibrin degradation products, respectively (lane 3). Fibrin degradation species were identified as reported in [32]. Graphs show quantitation of fibrin degradation products by densitometry, expressed as the percentage of the total protein quantified in each sample. (**e**) Cellular testisin facilitates plasmin(ogen)-dependent cell invasion through fibrin. TsWT, TsMut, and Ctl cells were plated onto fibrin or Matrigel-coated Transwells in serum-free medium with (+) or without (−) 250 µM TXA, and invasion towards 10% serum-containing media assessed. Representative images shown are stitched, whole-well scans of the underside of Transwells showing invaded cells stained with KwikDiff after 10 h invasion (4× original magnification). The two left panels (−TXA) show wells from 2 independent experiments; right panel (+TXA) shows wells from a representative experiment showing inhibition of testisin-mediated fibrin invasion. Images are representative of 3 independent experiments for both conditions. (**f**) Quantitation of cell invasion analyzed by manual cell counting of all invaded cells/membrane using Image J. TsWT invaded through fibrin ~13-fold faster than Ctl and TsMut cells, but invasion through Matrigel was not enhanced by expression of wildtype testisin. Data show average fold invasion relative to Ctl from 2–3 independent experiments, * *p* < 0.05; *** *p* < 0.005; **** *p* < 0.001; ns, non-significant.

The cell-based functional assay measuring fibrin generation revealed that in addition to accelerating fibrin generation, testisin-expressing TsWT cells hastened a decrease in turbidity (Figure 1), indicative of a more rapid cell-mediated dissolution of the formed fibrin polymers. To investigate whether a fibrinolytic mechanism was involved, we modified the fibrin generation assay to reach maximal fibrin polymerization within 2 h by adding exogenous active thrombin instead of prothrombin in the presence of fibrinogen and then monitored cell-based fibrinolysis over time. Plasmin is the major protease effector of fibrin degradation [37] and is produced from the zymogen plasminogen. For these assays, cells were grown in serum-containing media and washed once, leaving trace amounts of plasminogen associated with the cells [26]. Assays of cell-mediated fibrinolysis showed a more rapid loss of turbidity by TsWT cells compared to Ctl cells (Figure 4a), indicative of a testisin-mediated acceleration of fibrinolysis. A slower, delayed loss of turbidity was observed in Ctl cells, suggesting that endogenous fibrin degradation is less efficient in the absence of testisin. TsMut cells showed a similar delayed loss of turbidity as the Ctl cells (Figure 4a), demonstrating a requirement for testisin catalytic activity for the accelerated fibrinolytic activity of TsWT cells.

The presence of tranexamic acid (TXA, 250 µM), an anti-fibrinolytic agent and inhibitor of plasminogen activation [38], completely blocks both the accelerated fibrinolytic activity of TsWT cells and also the delayed fibrinolysis induced by TsMut and Ctl cells (Figure 4a, +TXA). Since TXA does not inhibit cell-expressed testisin activity (Figure 4b), these data indicate that the observed accelerated decrease in turbidity induced by TsWT expression is plasmin(ogen)-dependent. The average of 24 h endpoint turbidity measurements from multiple independent experiments shows that TsWT cells are significantly more effective at degrading fibrin than Ctl and TsMut cells by a process dependent on plasmin (Figure 4c).

Urea-solubilized lysates from the cell-based fibrinolysis assays collected at 24 h and analyzed by SDS-PAGE and Coomassie blue staining (Figure 4d) confirm that the loss of turbidity by TsWT cells is due to enhanced fibrin degradation. Compared to Ctl and TsMut, the TsWT cells show an increased loss of insoluble fibrin, as well as cleavage of the fibrin γ-γ dimers and the β-chain, resulting in the appearance of the fibrin degradation products γ-γ deg and β-deg, respectively (Figure 4d, lane 6 vs. lanes 4 and 8). These degradation products correspond to the fibrin degradation products produced as a result of plasmin cleavage (Figure 4d, lane 3), and their appearance is blocked by TXA (Figure 4d, lane 6 vs. lane 7), demonstrating plasmin(ogen) dependence.

### 2.6. Testisin Facilitates Cell Invasion through a Fibrin Matrix

In addition to fibrinolysis, the plasmin(ogen) system is involved in the control of cell invasion and extracellular matrix turnover [38,39]. The significance of membrane-anchored testisin in accelerating these plasmin-dependent activities was investigated by invasion assays through fibrin-coated Transwells. TsWT cells showed a significant ~13-fold increase in invasion through a fibrin matrix compared with Ctl or TsMut cells, which was inhibited in the presence of TXA (Figure 4e and Figure S4), demonstrating that invasion was plasmin-dependent. The average invasion from multiple independent experiments shows that TsWT cells are significantly more invasive through fibrin than Ctl and TsMut cells (Figure 4f). For comparison, TsWT, Ctl, and TsMut cells all invade through Transwells coated with Matrigel at similar efficiency (Figure 4f). Together, these data show that membrane-anchored testisin activates a plasmin-dependent pathway that enhances cell invasion through fibrin.

### 2.7. Plasminogen Accelerates Testisin-Mediated Cell-Associated Fibrinolysis

**Figure 5:**
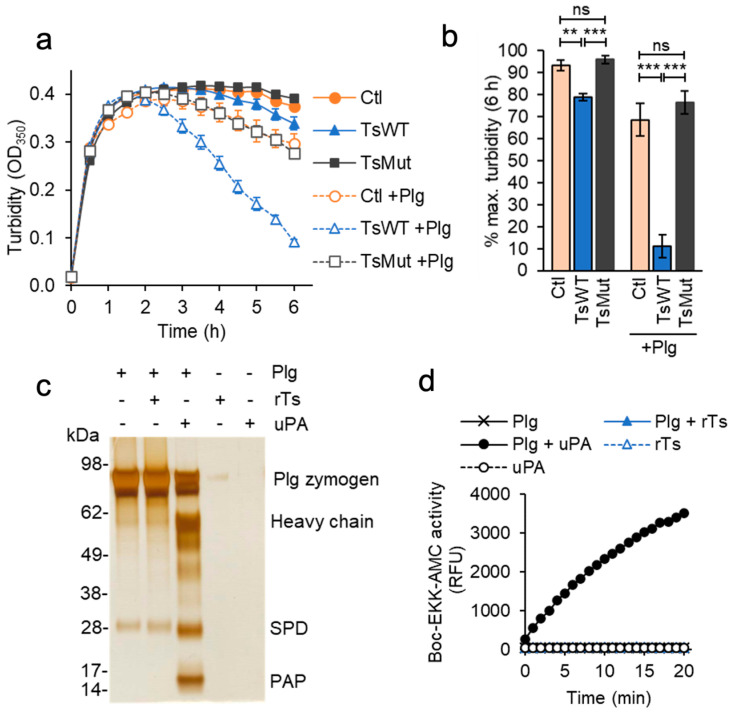
Testisin accelerates fibrin degradation but does not activate plasminogen directly. (**a**) Plasminogen accelerates TsWT-mediated fibrin degradation. Fibrin was generated by the addition of thrombin as in Figure 4a, in the absence (−) or presence (+) of 20 nM plasminogen (Plg). Graph shows average turbidity measured over 6 h from quadruplicate wells ± SEM and is representative of 3 independent experiments. (**b**) Average turbidity ±SEM at the 6 h endpoint from 3 independent experiments. ** *p* < 0.01; *** *p* < 0.005; ns, non-significant. (**c**) Unlike uPA, testisin does not activate the plasminogen zymogen in solution. 1.5 µM plasminogen (Plg) was incubated alone or with 30 nM rTs or uPA (50:1 zymogen-to-activating protease ratio) for 2 h at 37 °C, and reactions analyzed by reducing SDS-PAGE and silver staining. Gel shown is representative of 3 independent experiments and shows activation cleavage of the plasminogen zymogen to release the 55 kDa heavy chain, the 26 kDa serine protease domain (SPD), and the 8.2 kDa pre-activation peptide (PAP). (**d**) Plasmin activity assays after treatment of plasminogen with rTs or uPA as in (**c**). Reactions were diluted ~40-fold and incubated with 100 µM Boc-EKK-AMC plasmin substrate, and fluorescence monitored at 1 min for 20 min. Graph shows average relative fluorescence units (RFU) from duplicate wells and is representative of 4 independent experiments.

The assays of cell-mediated fibrinolysis above are conducted with trace amounts of plasminogen that remains associated with washed cells [26]. Increased levels of plasminogen might therefore be expected to further accelerate testisin-mediated enhancement of fibrinolysis. Indeed, the addition of exogenous plasminogen to the cell-based fibrinolysis assay dramatically accelerated the early phase of TsWT-mediated fibrinolysis, resulting in a nearly complete loss of fibrin by 6 h, compared to Ctl and TsMut cells (Figure 5a, +Plg). The average of endpoint turbidity measurements at 6 h from multiple independent experiments confirmed the significantly accelerated loss of fibrin by TsWT cells in the presence of added plasminogen compared with Ctl and TsMut cells (Figure 5b).

### 2.8. Testisin Does Not Activate Plasminogen Directly

To determine whether testisin was capable of activating plasminogen directly, recombinant testisin was incubated with the plasminogen zymogen in solution. Testisin was unable to cleave the 87 kDa plasminogen zymogen, whereas under the same conditions, recombinant uPA was able to catalyze the conversion of plasminogen zymogen to plasmin, indicated by the generation of the 55 kDa heavy chain, the 26 kDa serine protease domain (SPD), and the 8.2 kDa pre-activation peptide (PAP), which is released by plasmin cleavage [40] (Figure 5c). Moreover, the incubation of testisin with plasminogen did not generate plasmin activity, which occurs when plasminogen is incubated with recombinant active uPA, as measured by the fluorogenic Boc-EKK-AMC peptide (Figure 5d). These data indicate that testisin activates the fibrinolytic pathway upstream of plasminogen.

### 2.9. Membrane-Anchored Testisin Accelerates Fibrinolysis through Activation of Pro-uPA

**Figure 6:**
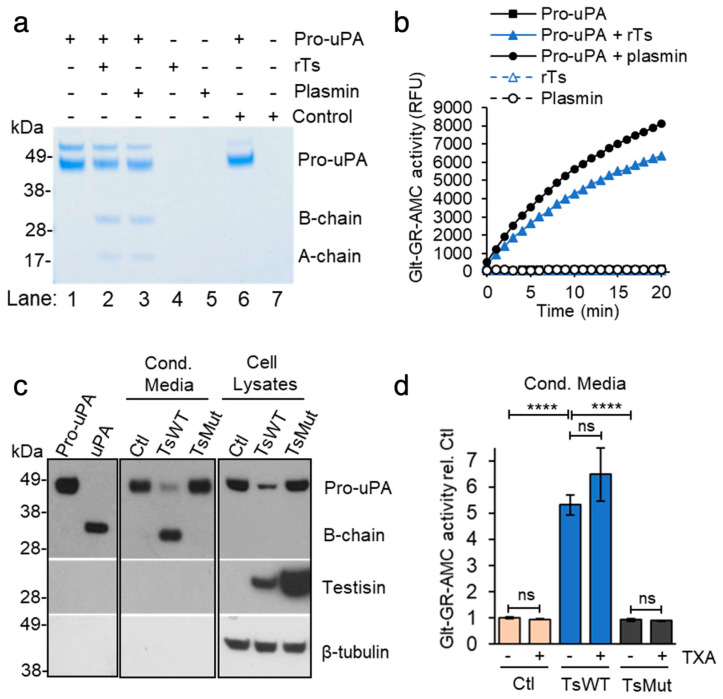
Cell-surface testisin induces pro-uPA activation. (**a**) Testisin directly activates the pro-uPA zymogen in solution. An amount of 1.5 µM pro-uPA was incubated alone or with 30 nM rTs or plasmin (50:1 zymogen-to-activating protease ratio) for 2 h at 37 °C, and reactions analyzed by reducing SDS-PAGE and Coomassie blue staining. Since the commercial preparation of rTs is in zymogen form and is activated by trace amounts of the metalloprotease thermolysin, reactions were incubated under identical conditions with equivalent final concentrations of thermolysin. Under these conditions, thermolysin does not cleave pro-uPA (*Cont*), demonstrating the pro-uPA cleavage is mediated specifically by testisin. Gel shown is representative of 4 independent experiments. The ~55 kDa band in the pro-uPA is likely a contaminating *Drosophila* protein that sometimes co-purifies with the recombinant pro-uPA produced in S2 cells as reported in [45]. (**b**) uPA activity assay of reactions in (**a**) using the Glt-GR-AMC uPA substrate. Graph shows average relative fluorescence units (RFU) from duplicate wells over 20 min and is representative of 3 independent experiments. (**c**) TsWT cells show increased activation of endogenous pro-uPA compared to Ctl and TsMut cells. Cell lysates and 10x-concentrated 24 h-conditioned media (Cond. Media) were immunoblotted for uPA, testisin, and β-tubulin loading control by stripping and reprobing. Purified recombinant pro-uPA (5 ng, 50 kDa) and active uPA proteins (10 ng, 33 kDa) were resolved in the first two lanes as molecular mass controls. Panels separating the blots indicate lanes in between samples that have been spliced out. For the uPA blot, Cond. Media is a shorter exposure than other two panels to enable comparison between Ctl, TsWT, and TsMut. Data are representative of 3 independent experiments. (**d**) TsWT-conditioned media has high uPA activity compared to that of Ctl and TsMut cells, which is not altered by the presence of the plasmin(ogen) inhibitor TXA. Conditioned media was collected from cells incubated for 24 h with (+) or without (−) 250 µM TXA and assayed for uPA activity using the Glt-GR-AMC uPA substrate. Graph shows average uPA activity ± SEM at 20 min from 3 independent experiments expressed relative to Ctl media, **** *p* < 0.001, ns, non-significant.

Plasmin is generated from plasminogen on cell surfaces predominantly through the action of uPA, which is a secreted protease, but it can be tethered to the cell surface by binding to its receptor, uPAR [41,42]. uPA is synthesized as a single-chain zymogen, pro-uPA, which is reciprocally activated by plasmin [43,44] by hydrolysis of the Lys158↓Ile159 bond, yielding two A and B chains, which remain covalently linked by a disulfide bond. To investigate whether testisin may catalyze the cleavage activation of pro-uPA directly, recombinant pro-uPA was incubated with recombinant testisin (rTs) and compared to cleavage by plasmin in a cell-free system. The cleavage of pro-uPA (49–50 kDa) by rTs generated peptide fragments similar to plasmin cleavage of pro-uPA, releasing the 33 kDa B-chain and the 20 kDa A-chain (Figure 6a, rTs versus plasmin, lanes 2 and 3). Pro-uPA cleavage by recombinant testisin generates proteolytically active uPA (Figure 6b), as measured using the Glt-GR-AMC substrate, which is based on the activating cleavage site in plasminogen [36]. These data indicated that the pro-uPA zymogen could be a testisin substrate on the cell surface.

We used an antibody that recognizes the uPA serine protease domain to determine the expression and activation status of endogenous uPA in Ctl, TsWT, and TsMut cells by SDS-PAGE under reducing conditions and immunoblot analysis (Figure 6c). An analysis of cell-associated uPA in lysates predominantly showed the pro-uPA zymogen form, with TsWT cells displaying reduced levels of the pro-uPA zymogen compared to Ctl and TsMut cells. At the same time, TsWT cells contained substantial levels of active uPA (B chain) in the conditioned media (Cond. Media), with a corresponding loss of zymogen pro-uPA compared to Ctl and TsMut cells (Figure 6c), suggesting increased activation of uPA in the presence of membrane-anchored testisin. An assay of the conditioned media for uPA activity showed substantially increased levels of uPA activity produced by TsWT cells, which is elevated over five-fold compared to Ctl and TsMut cells (Figure 6c). The plasmin(ogen) inhibitor TXA does not inhibit cell-associated pro-uPA activation by testisin (Figure 6d), excluding the possibility that testisin acts indirectly to stimulate plasmin generation, which could reciprocally activate pro-uPA on the cell surface. These data identify testisin as a cell-surface activator of pro-uPA that can induce cell-mediated plasmin(ogen)-dependent fibrinolysis.

## 3. Discussion

**Figure 7:**
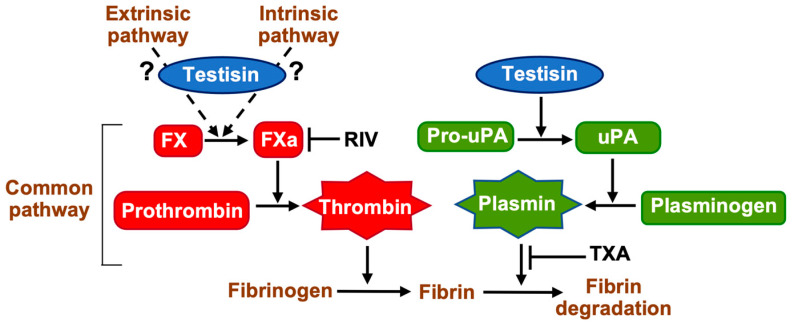
Zymogen activation pathways activated by membrane-anchored testisin that promote coagulation and fibrinolysis. GPI-anchored testisin promotes thrombin activation via stimulating the activation of the prothrombin activator FXa, which may occur via activation of an upstream protease of the intrinsic or extrinsic coagulation pathways or via a novel mechanism. Generation of active thrombin cleaves fibrinogen to induce its polymerization into fibrin. GPI-anchored testisin promotes plasmin generation via the activation of cell-surface pro-uPA, which then activates plasminogen to degrade polymerized fibrin. Shown also are inhibitors of FXa rivaroxaban (RIV) and plasminogen (TXA). Schematic is simplified for clarity.

Coagulation and fibrinolysis are characterized by the sequential, rapid, and highly localized activation of serine proteases. Serine proteases are produced as inactive zymogens whose activation is highly regulated, since inappropriate or unrestricted proteolysis has pathological consequences. Here, we have identified a unique role for the MASP testisin that intersects with extracellular fibrinolytic and coagulation pathways at cell surfaces. We found that testisin proteolytic activity can stimulate both FXa-dependent fibrin generation and accelerate plasminogen-dependent fibrinolysis (Figure 7), thus influencing pericellular hemostasis.

Testisin is a serine protease that remains linked to the cell surface through a GPI-anchor [4,5,6]. Our previous studies using a limited panel of peptide substrates and recombinant testisin determined that testisin possesses a trypsin-like substrate specificity with a preference for cleavage after P_1_-Arg over P_1_-Lys [46]. Testisin is expressed by endothelial cells of various vascular beds and during capillary morphogenesis [10,11,12]. When challenged with hormones that induce rapid angiogenesis of the corpus luteum, testisin-deficient mice display defective microvascular development, resulting in increased hemorrhages, implicating testisin in protecting endothelial barrier integrity during angiogenesis. Repair-associated angiogenesis is usually accompanied by both the presence of vascular leakage and fibrin deposition [18]. Fibrin deposition not only limits bleeding but also produces factors that stimulate angiogenesis and provide a scaffold for invading inflammatory, endothelial, and other cells during the healing process [18].

The serine protease thrombin plays a central role in the efficient control of hemorrhages. Using a reductionist approach to investigate the effect of testisin expression in cells that do not express endogenous testisin, we found that cells expressing active testisin on their cell surface could accelerate thrombin-dependent fibrin generation, which was validated by the visualization of fibrin(ogen) in urea-solubilized lysates. These cell-based fibrin generation assays were performed on single washed cells to ensure trace amounts of serum components remained present in the first instance, and subsequent experiments showed that cell-surface testisin does not activate the thrombin zymogen directly but stimulates prothrombin activation and fibrin generation through a pathway promoting FXa activation. FX is at the key intersection of the complex intrinsic and extrinsic coagulation cascades, and the testisin target responsible for promoting FXa activation is as yet unclear. Testisin may intersect with intrinsic or extrinsic pathways by activating an upstream coagulation protease zymogen that promotes the formation of Xase-activating complexes [21,22,47], or it may directly interact with FX when in complex with other cell surface proteins, as has been reported for cathepsin G activation of FX bound to CD11b/MAC-1 on monocytes [48].

Testisin-deficient mice have no developmental defects and no obvious defects in hemostasis when observed under unchallenged conditions [7,12], suggesting a post-natal role in angiogenesis. In adult organisms, the vascular tree is fully developed, and physiological angiogenesis is normally limited to the female reproductive system. However, the formation of new blood vessels becomes essential in adults during tissue repair after severe wounding or inflammation. Testisin may serve to maintain basic homeostasis or to re-establish vascular hemostasis after external challenge, such as occurs during tissue injury or inflammation. The restricted tissue expression of testisin and its localization to lipid rafts [4,5,6] suggests that its opposing activation of either pro-coagulative or pro-fibrinolytic pathways is likely to be highly dependent upon the local pericellular environment and the co-expression and localization of relevant cellular receptors. Positive and negative feedback loops interactively function to maintain the hemostatic balance to prevent unwanted bleeding and clotting. Testisin’s novel activities resemble the crosstalk between the intrinsic coagulation pathway with fibrinolytic proteases, where activated FXII stimulates coagulation but can also directly activate plasminogen [49,50], and plasmin is also reported to reciprocally activate FXII to FXIIa [51,52].

This study adds to the accumulating evidence that cross-activation between coagulation, fibrinolytic, and MASP cell-surface zymogen activation pathways exists. The human type II transmembrane serine protease (TTSP) hepsin has been shown to activate coagulation factor VII to initiate coagulation in vitro [53], and its knockdown in zebrafish models reduces factor VIIa plasma levels and leads to increased coagulation times [54]. The TTSP matriptase can be activated on the cell surface by both plasminogen [55] and coagulation protease FXa [56], and matriptase, hepsin, and TMPRSS4 are activators of pro-uPA on the cell surface [55,57,58,59]. Cell-surface plasmin generation is believed to be critically regulated by the binding of plasminogen to cell-surface receptors such as the integral membrane protein Plg-RK_T_ [60], allowing colocalization with uPA that is bound to its high-affinity cellular receptor uPAR [60,61]. This interaction of pro-uPA with cell-surface uPAR accelerates plasminogen activation and the reciprocal activation of pro-uPA [41,42]. In contrast to hepsin, matriptase, and TMPRSS4, which are anchored to the cell surface via N-terminal transmembrane domains, the GPI-anchorage of both testisin and uPAR likely serves to localize these proteins to cholesterol- and sphingolipid-rich lipid rafts and may enable a highly efficient activation of pro- uPA bound to uPAR by testisin that is dependent on their proximity on the cell surface.

Our cell-based activity assays clearly demonstrate high testisin activity in TsWT cells that is not observed in cells expressing the testisin active site mutant. However, the physiological protease(s) that induces testisin zymogen activation is not known. Though autoactivation has been reported for several TTSPs that contain modular domains in their stem regions [1], the simple structure of testisin resembles that of the only other GPI-anchored protease, prostasin, which is unable to autoactivate but is activated by the TTSP matriptase in a reciprocal zymogen activation complex [62]. Given the newly identified testisin activities reported here, it is of interest to determine whether specific coagulation or fibrinolytic enzymes can stimulate testisin’s activation.

Testisin is expressed in abundance on the sperm plasma membrane, where it has been shown to promote epidydimal sperm maturation and motility important for fertilizing ability [7]. Several of the coagulation and fibrinolytic proteases are found in semen [17,63], and uPA is present on the surface of human spermatozoa [64], suggesting a role for testisin in uPA-mediated sperm functions. In solid tumors, uPA and plasmin activities on the tumor cell surface promote tumor progression and metastasis [27], and aberrant expression of testisin could promote testisin-mediated uPA activation to enhance pro-metastatic tumor activities in vivo. Testisin overexpression is associated with ovarian cancer [14,15,65]. Fibrin is a significant component of ovarian tumor pathology [66], and ovarian cancer-associated ascites fluid is rich in cross-linked fibrin and fibrin degradation products. High levels of the characteristic fibrin degradation product D-dimer in the plasma of ovarian cancer patients is predictive of poor prognosis [67] and is associated with increased risk for the development of venous thromboembolism [67,68]. Active testisin on the endothelial cell surface would contribute to fibrin formation and uPA/plasmin-mediated fibrin degradation required for angiogenic processes of cell migration, reorganization, and vessel stabilization [18,69], essential mechanisms for the repair of blood vessels after acute injury or chronic inflammation. How these testisin-stimulated proteolytic activities regulate the localized hemostatic balance on cell surfaces in ischemic diseases and in cancer remain an area of considerable interest.

## 4. Materials and Methods

### 4.1. Reagents

Human fibrinogen (plasminogen, von Willebrand factor, and fibronectin-depleted) and human zymogens Glu-plasminogen, α-thrombin, prothrombin, factor X zymogen, and activated factor Xa (all >95% purity) were purchased from Enzyme Research Laboratories, South Bend, IN, USA. Human plasmin and tranexamic acid (TXA) were purchased from MilliporeSigma, Burlington, MA, USA. Recombinant human pro-uPA zymogen [70] was kindly provided M. Ploug (Finsen Laboratory, Copenhagen, Denmark). Recombinant human uPA and recombinant mouse testisin proteins were from R&D Systems, Minneapolis, MN, USA. Fluorogenic substrates for thrombin (thrombin substrate III, Bz-FVR-AMC), FXa (Boc-IEGR-AMC [36]), uPA (Glt-GR-AMC [36]), plasmin (Boc-EEK-AMC [71]), and trypsin-like (Boc-QAR-AMC) proteases were from BACHEM, Bubendorf, Switzerland. Rivaroxaban (BAY 59-7939) was from Thermo Fisher Scientific, Waltham, MA, USA. Antibodies used were mouse anti-testisin antibody (MAbD9.1) [12], mouse anti-uPA antibody (IM15L, MilliporeSigma, Burlington, MA, USA), and rabbit anti-β-tubulin antibody (Santa-Cruz Biotechnology, Dallas, TX, USA). Secondary antibodies were goat anti-mouse-HRP, mouse anti-rabbit-HRP (Jackson ImmunoResearch, West Grove, PA, USA), and donkey anti-sheep-HRP (R&D Systems, Minneapolis, MN, USA).

### 4.2. Cell Culture

ES-2 cells stably transduced with lentiviral plasmids expressing full-length GPI-anchored human testisin (TsWT), the catalytically inactive testisin mutant (TsMut) in which the active site serine (Ser^238^) is replaced with alanine or vector alone (Ctl) have been described previously [14]. Cells were routinely cultured in DMEM containing 10% FBS (Complete Media) and passaged using the non-enzymatic reagent Versene (Gibco, Thermo Fisher Scientific, Waltham, MA, USA). For cell-based fibrin generation assays, cells were plated in 96-well trays and cultured in Complete Media prior to the assay. Cells were washed once in serum-free Opti-MEM (Gibco, Thermo Fisher Scientific, Waltham, MA, USA) supplemented with 5 mM CaCl_2_ and 20 mM HEPES pH 7.4, hereafter referred to as SFM + CaCl_2_, before replacement with SFM + CaCl_2_ containing fibrinogen as described below. This brief wash appears to preserve trace amounts of serum-derived protease zymogens [26].

### 4.3. Cell-Based Fibrin Generation Assays

Cells (plated at 5 × 10^4^ cells/well) were cultured for 48 h in 96-well trays and washed once in SFM + CaCl_2_ and media replaced with 100 µL/well of SFM + CaCl_2_ containing 1 mg/mL fibrinogen and 10 nM prothrombin, where indicated. In some experiments, the FXa inhibitor rivaroxaban at 20 or 50 nM final concentration was also included. Plates were incubated at 37 °C, and turbidity at OD_350_ nm measured every 20–30 min using a FlexStation3^®^ microplate reader (Molecular Devices, San Jose, CA, USA). Average turbidity for each condition/timepoint was determined from 3–4 replicate wells after subtracting the turbidity of wells containing fibrinogen in SFM + CaCl_2_ alone.

### 4.4. Cell-Based Fibrinolysis Assays

Cells (plated at 2 × 10^4^ cells/well) were cultured overnight in 96-well trays and washed once in SFM + CaCl_2_ and media replaced with 50 µL of SFM + CaCl_2_ containing 2 mg/mL fibrinogen. Fifty µL of human α-thrombin at 0.04 U/mL was then added to induce fibrin polymerization. Trays were incubated at 37 °C, and turbidity (OD_350_) monitored over time, with maximal turbidity being reached at ~2–3 h, which is followed by a decrease in turbidity representative of fibrinolysis. In some experiments, the plasmin(ogen) inhibitor tranexamic acid (TXA, 250 µM) or plasminogen (20 nM) were also included. Average turbidity from 3–4 replicate wells was calculated after subtracting the turbidity of wells containing SFM + CaCl_2_ alone. Averaged data from multiple experiments are expressed as % maximal turbidity due to some variability in the maximal turbidity reached after the addition of thrombin between individual experiments.

### 4.5. Detection of Fibrin(ogen) by SDS-PAGE

At indicated time points, 100 µL of 2X urea lysis buffer (8 M Urea, 10% SDS, and 5% β-mercaptoethanol) was added per well, and samples solubilized at room temperature overnight. Equivalent volumes of lysate were diluted in reducing NuPAGE^TM^ LDS sample buffer (Thermo Fisher Scientific, Waltham, MA, USA), heated to 70 °C for 10 min, and analyzed by SDS-PAGE and Coomassie blue staining (Gel Code Blue, Thermo Fisher Scientific, Waltham, MA, USA). Densitometry analysis of destained gels was performed using ImageJ software (NIH) version 1.53k, and quantified signals of protein bands of interest expressed as % total fibrin(ogen) bands per sample.

### 4.6. Cell-Free Fluorogenic Peptide Activity Assays

Recombinant human testisin (R&D Systems, Minneapolis, MN, USA) was activated according to the manufacturer’s instructions using the metalloprotease thermolysin, which is then inhibited by the addition of 1,10-phenanthroline. In all assays conducted using recombinant testisin, control reactions were set up containing the equivalent volume of reaction buffer containing thermolysin/phenanthroline alone (denoted “Control” when shown in figures), which showed no activity on any substrate or protease zymogen tested. For direct assessment of substrate cleavage, 1–10 nM protease as indicated was incubated with 100 µM fluorogenic peptide substrates in 100 µL assay buffer (50 mM Tris, 150 mM NaCl, 10 mM CaCl_2_) at 37 °C, and emitted fluorescence measured at 1 min intervals at Ex370 nM/Em450 nM in a FlexStation 3^®^ microplate reader (Molecular Devices, San Jose, CA, USA). Activity is expressed as relative fluorescence units (RFU) from duplicate wells. In some experiments, rivaroxaban (50 nM) was preincubated in the reactions for 10 min at room temperature prior to adding substrates.

### 4.7. Cell-Based Fluorogenic Peptide Activity Assays

Confluent ES-2 cells plated in 96-well black-walled clear bottom trays were washed once in SFM + CaCl_2_, and media replaced with SFM + CaCl_2_ containing 100 µM peptide substrates and 1 mg/mL fibrinogen, with the addition of protease zymogens or inhibitors as indicated. Cells were then incubated at 37 °C, and emitted fluorescence measured at 20 or 30 min intervals at for the indicated times. Fluorescence values from wells containing substrate alone with 1 mg/mL fibrinogen were subtracted from readings. Representative curves are presented as mean ± SEM from 3–4 wells/condition, and activity detected at 3 h was averaged from multiple independent experiments as indicated.

### 4.8. Activation of Protease Zymogens by Recombinant Testisin (rTs)

To assess activation of FX, plasminogen, and pro-uPA, protease zymogens (1.5 µM) were incubated with rTs (30 nM or 60 nM) in 30 µL assay buffer (50 mM Tris, 150 mM NaCl, 10 mM CaCl_2_). Positive control reactions contained 30 nM known activating proteases (uPA for plasminogen, plasmin for pro-uPA). Reactions were incubated at 37 °C for 2 h, after which half of the reactions were analyzed by SDS-PAGE, followed by silver staining using the Pierce Silver Stain Kit (Thermo Fisher Scientific, Waltham, MA, USA) or Coomassie blue. Activity assays were performed on 50 µL duplicate samples after diluting reactions 1:37.5 in assay buffer and adding 50 µL of 200 µM peptide substrates (final concentration of initial protease zymogens of 40 nM).

### 4.9. Cellular Pro-uPA Activation

For detection of endogenous pro-uPA produced by ES-2 cell lines, cells (8 × 10^5^ cells/well) were plated in a 12-well tray in Complete Media and allowed to adhere overnight. Cells were washed once, media replaced with SFM + CaCl_2_ with or without 250 µM TXA, and cell lysates and conditioned media collected 24 h later. After centrifugation to remove any cell debris, conditioned media were concentrated 10-fold using 0.5 mL protein concentrators (3000 Da MW cutoff, Pierce, Thermo Fisher Scientific, Waltham, MA, USA). Equal volumes of concentrated conditioned media and equal concentrations of protein lysates were analyzed by reducing SDS-PAGE and western blot analysis using mouse anti-uPA antibody (IM15L). For assessment of uPA activity, 20 µL of unconcentrated conditioned media was diluted in assay buffer and 100 µM uPA substrate Glt-GR-AMC, and fluorescence measured at 1 min intervals at Ex370 nM/Em450 nM over 20 min.

### 4.10. Cell Lysis and Immunoblot Analysis

Cell lysates were prepared in lysis buffer (0.5% Triton, 0.5% NP40, 50 mM HEPES, 150 mM NaCl, pH 7.3) containing cOmplete™, Mini Protease Inhibitor Cocktail (Roche, MilliporeSigma, Burlington, MA, USA). Protein concentrations were determined using Protein Assay Dye (Bio-Rad, Hercules, CA, USA). Samples containing equal protein were prepared in reducing NuPAGE LDS sample buffer (Thermo Fisher Scientific, Waltham, MA, USA) heated at 70 °C for 10 min. Samples were resolved on 4–12% NuPAGE SDS-PAGE gels and transferred to PVDF membrane (MilliporeSigma, Burlington, MA, USA) according to standard techniques. After incubation with primary and secondary antibodies, immunoreactive bands were visualized using SuperSignal West Pico Chemiluminescent Substrate (Pierce, Thermo Fisher Scientific, Waltham, MA, USA) and exposure to film.

### 4.11. Transwell Invasion Assays

Six-and-a-half mm polycarbonate Transwell^®^ filters with 8 µm pores (Corning, Thermo Fisher Scientific, Waltham, MA, USA) were coated with 50 µL fibrin (1 mg/mL fibrinogen, 0.02 U/mL thrombin) or 1 mg/mL Matrigel^®^ (Corning, Thermo Fisher Scientific, Waltham, MA, USA), which were allowed to solidify at 37 °C for 1 h. Cell lines were collected and washed once in serum-free (SF)-DMEM, and 5 × 10^4^ cells in 100 µL SF-DMEM plated into upper chambers in duplicate; 0.5 mL DMEM containing 10% FBS was placed into lower chambers as a chemoattractant, and cells were incubated at 37 °C for 10–12 h. In some experiments, the plasmin inhibitor tranexamic acid (TXA, 250 µM) was included in the cell suspensions added to the upper chamber. At the endpoint, top chambers were washed and cleaned with a cotton swab, and invaded cells on the underside of the Transwell filter were stained with Kwik-Diff^TM^ (Epredia, Thermo Fisher Scientific, Waltham, MA, USA) as per the manufacturer’s instructions. Filters were imaged using the EVOS FL Auto 2 Imaging system (Invitrogen, Thermo Fisher Scientific, Waltham, MA, USA), and all invaded cells/Transwell were counted using the multipoint tool on ImageJ software (NIH) version 1.52q.

### 4.12. Data Analysis and Statistics

Data are expressed as means ± SEM. Statistical analyses were performed using the two-tailed unpaired *t* test to determine statistically significant differences between test conditions. A threshold of *p* < 0.05 was considered significant.

## Data Availability

All data are available within this manuscript and the Supplementary Materials.

## Supplementary Materials

The supporting information can be downloaded at: https://www.mdpi.com/article/10.3390/ijms24119306/s1.
