# Supplementary material for: Intersection of Coagulation and Fibrinolysis by the Glycosylphosphatidylinositol (GPI)-Anchored Serine Protease Testisin
Source: Int J Mol Sci. 2023 May 26;24(11):9306. doi: 10.3390/ijms24119306 (PMC10252689; doi:10.3390/ijms24119306)
Supplement: Supplementary file 1 [file ijms-24-09306-s001.zip › ijms-2370732-supplementary.pdf]

SUPPLEMENTARY MATERIALS

Intersection of coagulation and fibrinolysis by the glycosylphosphatidylinositol (GPI)-anchored serine protease testisin.

Marguerite S. Buzza, Nisha R. Pawar, Amando A. Strong and Toni M. Antalis.

This file includes: Supplementary Figures S1-S4

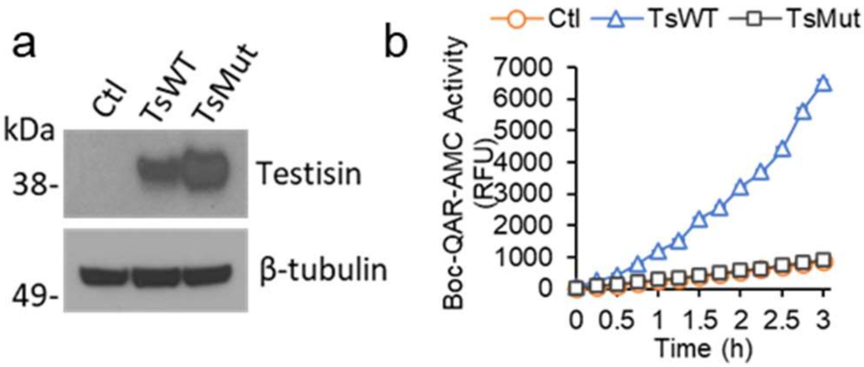

Figure S1. Testisin expression and activity in TsWT, TsMut and Ctl cells. (a) Whole cell lysates were collected from TsWT (wildtype testisin), TsMut (active site mutant testisin) and Ctl (vector alone) cells and analyzed by immunoblot for testisin protein expression. Blots were reprobed with β-tubulin as a control for protein loading. **(b)** High testisin activity in TsWT cells compared to TsMut and Ctl cells as measured using the fluorogenic substrate Boc-QAR-AMC. Assay was performed in the presence of 1 mg/ml fibrinogen to replicate conditions used in fibrin generation assays. Graph is a representative experiment showing the mean ± SEM from triplicate wells. Average relative activities of these lines from multiple independent experiments are shown in Figures 3h and 4b.

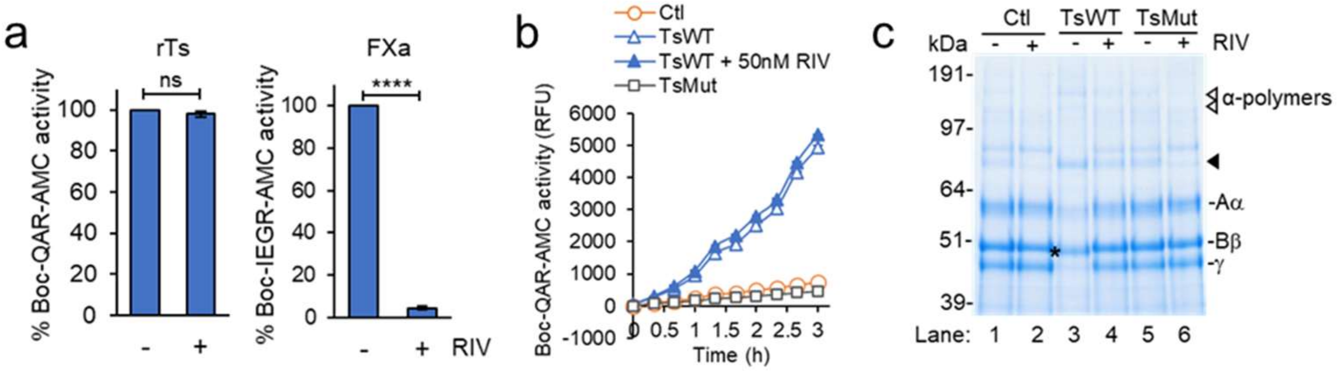

Figure S2. Testisin mediated fibrin generation is inhibited by the FXa inhibitor rivaroxaban, which does not inhibit testisin's activity. **(a)** The activity of recombinant testisin (rTs) is not inhibited by rivaroxaban. 5 nM rTs or 1 nM FXa were pre-incubated with (+) or without (-) a 10 fold molar excess of rivaroxaban (RIV) for 10 min, and then assayed for testisin activity using Boc-QAR-AMC (rTs) or FXa activity using Boc-IEGR-AMC (FXa). Data is expressed as average % activity relative to enzymatic activity in the absence of RIV ±SEM at 20 min (rTs) or 10 min (FXa) after adding substrate, from 2 independent experiments. ****p<0.001, ns, non-significant. **(b)** Rivaroxaban does not inhibit cell-expressed testisin. Testisin activity was assayed in Ctl, TsMut, TsWT and TsWT cells in the presence of 1 mg/ml fibrinogen +/- 50 nM rivaroxaban using the Boc-QAR-AMC substrate over 3 h. Graph is representative of 3 independent experiments, mean ± SEM from triplicate wells. **(c)** Urea solubilized lysates of cells incubated in the presence of 1 mg/ml fibrinogen and 10 nM prothrombin at 3 h in the presence (+) or absence (-) of 50 nM rivaroxaban (RIV). Rivaroxaban inhibits TsWT mediated fibrin generation, as indicated by the loss of testisin-induced γ-γ dimers (black arrowhead) and thrombin cleaved β-chain (*), and the increased levels of the monomer Aα and γ-chains (*lane 3 vs lane 4*).

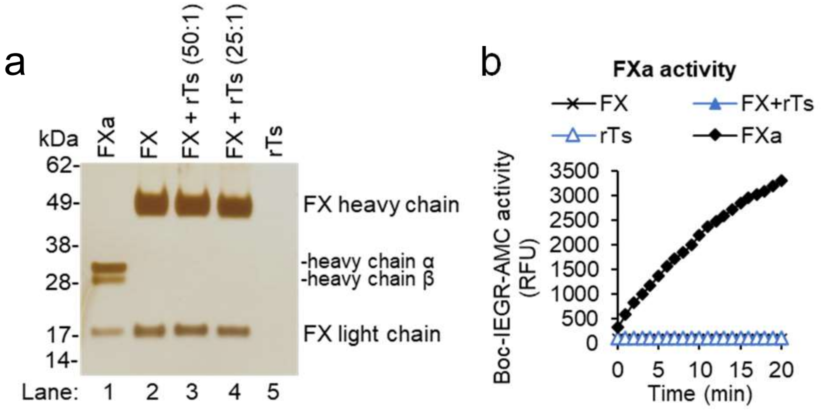

Figure S3. Testisin does not directly activate the FX zymogen. (a) rTs does not cleave the FX zymogen in solution. 1.5 μM FX zymogen was incubated in solution alone (FX) or with 30 nM rTs (50:1 molar ratio, *lane 3*) or 60 nM rTs (25:1 molar ratio, *lane 4*) for 2 hours at 37°C and analyzed by reducing SDS-PAGE and silver-staining. *Lane 1* contains FXa for a molecular mass reference. Activation cleavage of FX produces a heavy chain α derived from cleavage at the FXa zymogen activation site and a smaller heavy chain β generated by auto-proteolytic cleavage. Gel is representative of 3 independent experiments. **(b)** Activity on the FXa substrate Boc-IEGR-AMC after preincubation of FX with rTs (25:1) for 2 hrs as in (a). Positive control reactions for substrate cleavage contained 5 nM active FXa. Graph is representative of 3 independent experiments.

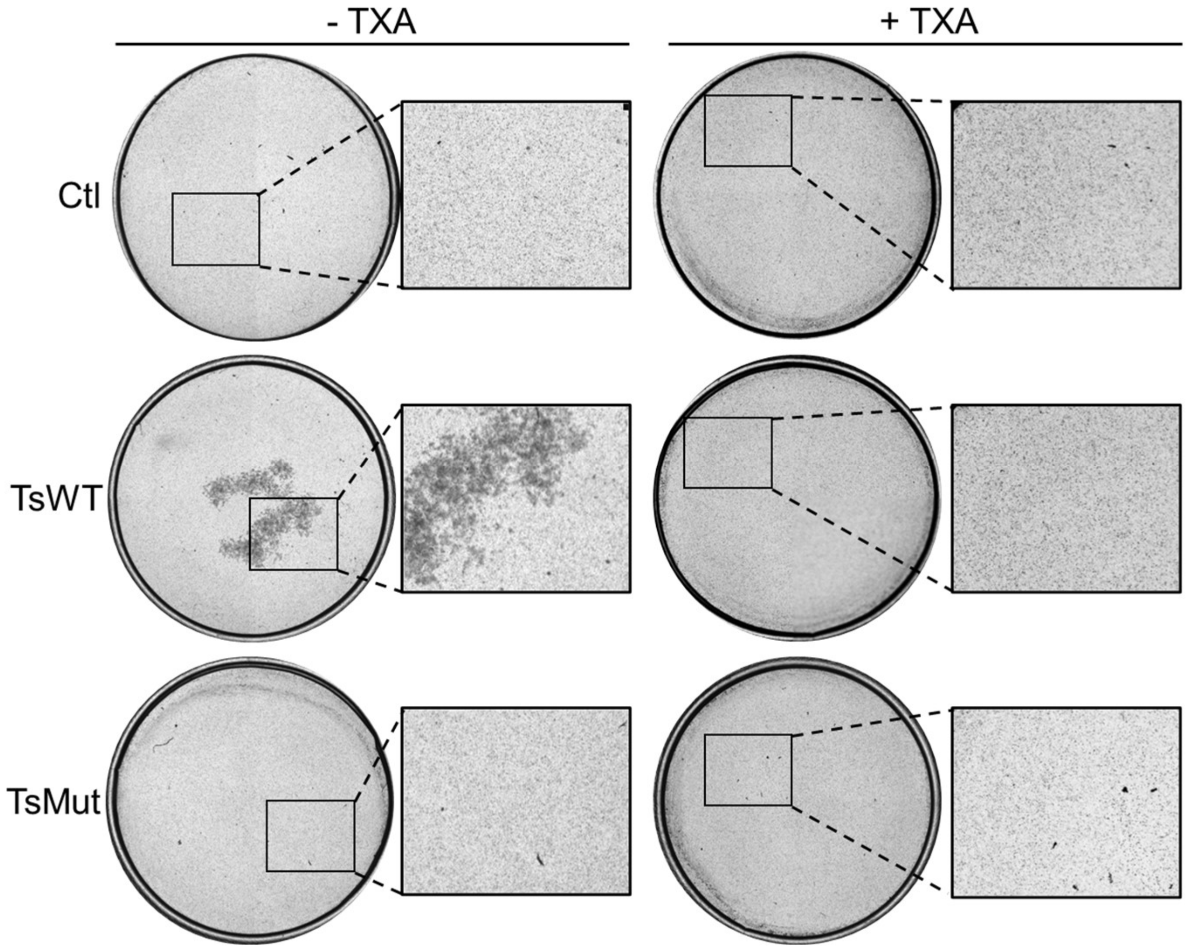

Figure S4. Testisin expression accelerates plasmin(ogen)-dependent cell invasion through fibrin. Enlargement of transwell invasion images shown in Figure 4e, left (-TXA) and right panels (+TXA). Circular images are stitched together scans of the underside of Transwells, showing invaded cells stained with Quikdiff after 10 h invasion through fibrin. Boxes outline enlarged areas for visualization of invaded cells.
